# B Cell Activating Factor (BAFF) and T Cells Cooperate to Breach B Cell Tolerance in Lupus-Prone New Zealand Black (NZB) Mice

**DOI:** 10.1371/journal.pone.0011691

**Published:** 2010-07-21

**Authors:** Nan-Hua Chang, Yui-Ho Cheung, Christina Loh, Evelyn Pau, Valerie Roy, Yong-Chun Cai, Joan Wither

**Affiliations:** 1 Arthritis Centre of Excellence, Toronto Western Research Institute, Toronto, Ontario, Canada; 2 Department of Immunology, University of Toronto, Toronto, Ontario, Canada; 3 Department of Medicine, University Health Network, Toronto, Ontario, Canada; University Paris Sud, France

## Abstract

The presence of autoantibodies in New Zealand Black (NZB) mice suggests a B cell tolerance defect however the nature of this defect is unknown. To determine whether defects in B cell anergy contribute to the autoimmune phenotype in NZB mice, soluble hen egg lysozyme (sHEL) and anti-HEL Ig transgenes were bred onto the NZB background to generate double transgenic (dTg) mice. NZB dTg mice had elevated levels of anti-HEL antibodies, despite apparently normal B cell functional anergy *in-vitro*. NZB dTg B cells also demonstrated increased survival and abnormal entry into the follicular compartment following transfer into sHEL mice. Since this process is dependent on BAFF, BAFF serum and mRNA levels were assessed and were found to be significantly elevated in NZB dTg mice. Treatment of NZB sHEL recipient mice with TACI-Ig reduced NZB dTg B cell survival following adoptive transfer, confirming the role of BAFF in this process. Although NZB mice had modestly elevated BAFF, the enhanced NZB B cell survival response appeared to result from an altered response to BAFF. In contrast, T cell blockade had a minimal effect on B cell survival, but inhibited anti-HEL antibody production. The findings suggest that the modest BAFF elevations in NZB mice are sufficient to perturb B cell tolerance, particularly when acting in concert with B cell functional abnormalities and T cell help.

## Introduction

Systemic lupus erythematosus (SLE) is a multi-system autoimmune disease characterized by production of pathogenic anti-nuclear antibodies (ANA), resulting in the formation of immune complexes. The production of ANA suggests a loss of tolerance to nuclear antigens, however the precise defects leading to this breach remain unclear. NZB mice develop high titer anti-RBC and -ssDNA antibodies (Abs) leading to a Coomb's positive hemolytic anemia and mild glomerulonephritis [1]. One of the characteristics of these mice is polyclonal B cell activation, similar to that observed in human SLE, suggesting that characterization of the defects in these mice may be particularly relevant to the human disease [2]-[7].

Induction and maintenance of B cell tolerance involves a series of checkpoints acting through multiple phases of B cell development [8]-[10]. We have previously shown that deletion of B cell receptor (BCR)-engaged early transitional (T1) B cells is defective in NZB mice [11]. Since normally these self-reactive B cells are rendered anergic and fail to enter the mature re-circulating B cell pool [12]–[14], the presence of activated auto-reactive B cells in the peripheral repertoire of these mice suggests that there are additional B cell tolerance defects.

Entry of transitional B cells into the mature B cell re-circulating pool is critically-dependent upon B cell-activating factor (BAFF) [15]. Patients with SLE exhibit elevated levels of serum BAFF [16]–[18] and over-expression of BAFF in BAFF transgenic (Tg) mice leads to development of a lupus-like phenotype [19], [20]. Based upon findings in these mice, it has been proposed that increased levels of BAFF breach B cell tolerance in lupus by enhancing survival of self-reactive B cells, thus allowing their abnormal entry into the mature follicular compartment where they can receive T cell help [21]. However, it is unclear whether the levels in lupus patients, which are considerably less than seen in BAFF transgenic mice, are sufficient to produce these abnormalities.

Here, we have investigated the induction and maintenance of B cell anergy in NZB mice. Anti-hen egg white lysozyme (HEL) Ig and soluble HEL (sHEL) transgenes (Tg) were crossed onto the NZB background and intercrossed to produce double Tg (dTg) mice, a well-characterized model of B cell anergy [22]. Although B cells in NZB dTg mice appeared phenotypically and functionally anergic, these mice produced high serum levels of anti-HEL Ab, suggesting that B cell anergy had been overcome. Using adoptive transfer experiments, we show that NZB dTg B cells demonstrate enhanced survival and abnormal entry into the follicular compartment, similar to that seen for BAFF Tg mice. We further demonstrate that the increased survival of transferred dTg B cells is BAFF-dependent and that BAFF cooperates with T cells to breach tolerance in these mice. Although NZB mice demonstrated modest increases in BAFF, the increased survival of NZB dTg B cells appeared to arise from a heightened response to BAFF.

## Results

### NZB dTg mice produce anti-HEL autoAb

**Figure 1:**
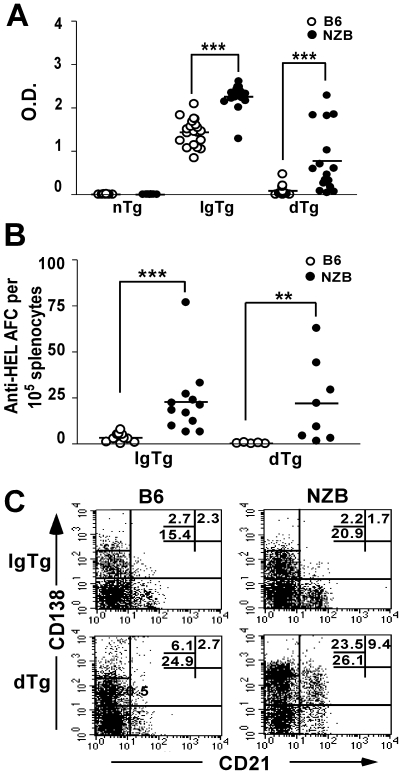
Breach of B cell anergy in NZB dTg mice. (A) Serum levels of IgM^a^ anti-HEL antibodies in 8–12 wk old B6 and NZB non-Tg (nTg), IgTg and dTg mice. (B) Presence of anti-HEL Ab-producing cells in NZB dTg mice. The number of anti-HEL Ab-forming cells was determined by ELISpot in 8-12 wk old B6 and NZB IgTg and dTg mice. Horizontal lines represent the mean for each group examined. (C) Presence of an increased proportion of early (CD21^+^CD138^int^) and late (CD138^high^) pre-plasma cells in NZB dTg mice. Bone marrow cells from 8 wk old B6 and NZB IgTg and dTg mice were stained with anti-B220, -CD138, and -CD21 Abs and analyzed by flow cytometry. Dot plots are gated on the B220^+^ population and numbers indicate the percentage of gated cells in each region. Asterisks indicate the significance level for comparison between B6 and NZB mice as determined by the Mann-Whitney test: * p<0.05, ** p<0.005, *** p<0.0005.

On the B6 genetic background, introduction of the anti-HEL IgTg results in high serum levels of anti-HEL Ab, which are absent in B6 dTg mice as a consequence of B cell anergy [22]. NZB IgTg mice produce higher levels of anti-HEL Ab than their B6 IgTg counterparts (Figure 1A), which we have previously shown to reflect the relative inability of the anti-HEL Ig transgene to normalize the polyclonal B cell activation phenotype in NZB mice [23]. NZB dTg mice produced significant levels of HEL-specific IgM^a^ Ab, which in some mice approached the levels seen in B6 IgTg mice (Figure 1A). Consistent with the elevated serum anti-HEL Ab levels, increased numbers of anti-HEL Ab-producing cells were detected in the spleens (Figure 1B) and bone marrow (data not shown) of NZB dTg mice. Immunofluorescent microscopy of frozen splenic sections also revealed HEL-positive cells within the marginal zone, red-pulp, bridging channels, and T cell zone of NZB, but not B6 dTg mice. These cells were IgM^abright^ suggesting that they were plasmablasts (data not shown).

As regulation of plasma cell differentiation for self-tolerant B lymphocytes occurs at the transition from the early to late pre-plasma cell stage [24], we examined these cell populations in NZB dTg mice. As shown in Figure 1C, early pre-plasma cells (CD21^−/+^CD138^int^) were found in the bone marrow of both B6 and NZB dTg mice, but NZB dTg mice had a significantly higher proportion of these cells. In addition, a marked increase in CD138^high^ late pre-plasma cells was seen in NZB dTg mice. Similar findings were observed for IgM^a+^ or HEL-binding B cells. Thus, the immune mechanisms preventing differentiation of anergic B cells to late pre-plasma and Ab-forming cells are defective in NZB mice.

### NZB dTg B cells are phenotypically and functionally anergic

As shown in Table 1, over 99% of B cells in both B6 and NZB IgTg mice expressed the IgM^a^ transgenic heavy chain and bound to HEL. NZB dTg mice had similar reductions in B cell numbers and cell surface IgM^a^ expression to B6 dTg mice, consistent with an anergic phenotype [25], [26] (Table 1). Down-regulation of cell surface IgM^a^ in dTg mice results from chronic antigen-engagement and reflects both the serum concentration of HEL and signaling capacity of the B cell [26]–[28]. Serum from NZB and B6 dTg mice down-regulated IgM^a^ equivalently on IgTg B cells *in-vitro* with both B6 and NZB IgTg B cells demonstrating similarly reduced levels of IgM^a^. Thus, both the serum concentration of HEL and signaling threshold for IgM^a^ down-regulation appear to be the same in B6 and NZB dTg mice.

**Table 1:** Comparisons of splenic populations in B6 and NZB nTg, IgTg, and dTg mice.

	B6			NZB		
	nTg (N = 4)	IgTg (N = 11)	dTg (N = 7)	nTg (N = 4)	IgTg (N = 17)	dTg (N = 11)
# splenocytes per spleen (x 10^6^)	32.44±6.84	38.76±12.99	31.54±9.90	50.88±7.16 *	46.31±12.34	35.4±14.44
# B220^+^ cells per spleen (x 10^6^)	20.28±3.35	22.47±8.20	**11.26±3.53**	21.65±7.85	21.65±7.85	**8.62±3.08**
% B220^+^	62.95±3.16	57.26±3.77	**35.64±4.53**	41.15±5.32 *	46.37±8.17	25.96±8.40 **
% IgM^a+^	N.D.	99.74±0.37	**96.13±0.73**	N.D.	99.47±0.36	91.60±2.77 ***
IgM^a^ MFI	N.D.	1428±276.7	**142.4±54.51**	N.D.	860.8±155.8 ***	**115.5±83.02**
% IgM^a+^ HEL^+^	N.D.	97.21±1.90	**88.36±4.02**	N.D.	97.74±2.00	**87.48±4.31**
% IgM^a+^ HEL^low/−^	N.D.	2.90±2.12	**7.78±3.83**	N.D.	1.73±1.92	4.23±2.10 *
% IgM^a−^ HEL^+^	N.D.	0.018±0.033	**0.87±0.52**	N.D.	0.047±0.061	3.02±1.73 **
% IgM^a−^ HEL^low/−^	N.D.	0.18±0.30	**2.57±0.70**	N.D.	0.44±0.31	4.83±1.97 **
% T1 (CD21^low^ CD24^hi^)	N.D.	19.03±5.31	**39.26±8.84**	N.D.	13.98±5.17	18.86±6.06 ***
% T2 (CD21^int^ CD24^hi^)	N.D.	6.84±3.03	**11.36±3.73**	N.D.	7.33±4.62	18.37±7.48 *
% MZ/MZP (CD21^hi^ CD24^hi^)	N.D.	**18.18±3.13**	7.65±3.04	N.D.	33.85±12.87 *	11.45±4.96
% Fo (CD21^int^ CD24^int^)	N.D.	**50.18±6.47**	**29.93±8.96**	N.D.	34.84±7.46 **	41.21±8.08 *

Results are mean ± SD. Numbers in brackets denote the number of 2 to 3 mo old female mice examined in each group. The proportion of anti-IgM^a^- and/or HEL-staining and B cell subsets are expressed as a percentage of the B220^+^ population. Asterisks indicate the significance level for comparison of NZB non-Tg with B6 non-Tg, NZB IgTg with B6 IgTg, or NZB dTg with B6 dTg mice, as determined by the Mann-Whitney test: * p<0.05, ** p<0.005, *** p<0.0005. Numbers are shown in bold if there is a significant difference (p<0.05) between IgTg and dTg of the same strain. N.D. not done. B cell subsets were identified using combinations of anti-B220, -CD24 and -CD21. T1  =  early transitional, T2  =  late transitional, MZ  =  marginal zone, FO  =  follicular, MZ precursor  =  MZP.

Chronic antigen-engagement of B cells with HEL in B6 dTg mice results in up-regulation of *Rag* expression leading to attempted receptor editing [10], [13]. When these endogenous light chains impair HEL binding they can be detected as IgM^a+^HEL^low/−^ cells, whose cell surface expression of IgM^a^ is higher than anergic dTg B cells [10]. Consistent with previous reports, there was an increased proportion of IgM^a+^HEL^low/−^ B cells in B6 dTg as compared to B6 IgTg mice (Table 1). The proportion of these cells was significantly less in NZB dTg mice, suggesting that there is reduced induction of receptor editing and/or production of effectively competing light chains in these mice.

**Figure 2:**
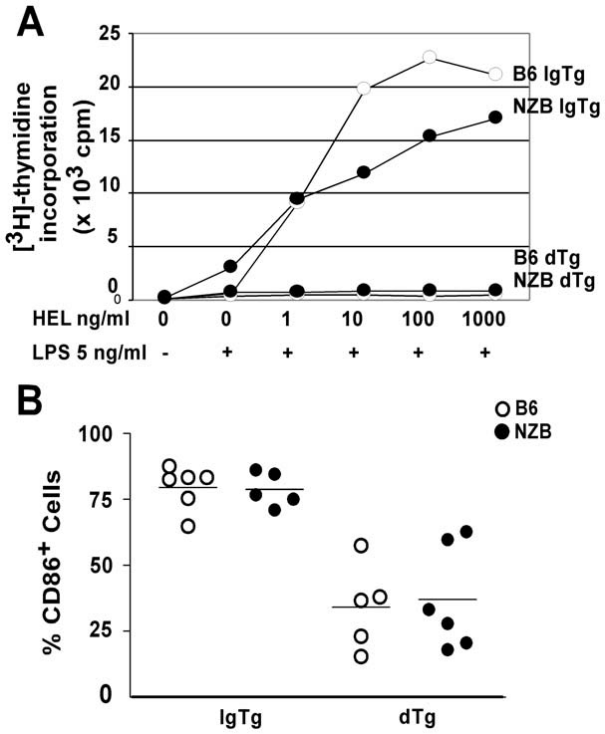
NZB dTg B cells appear functionally anergic *in-vitro*. (A) Sorted B cells from IgTg and dTg B6 or NZB mice were stimulated *in-vitro* with increasing concentrations of HEL (0 to 1 µg/ml) together with a submitogenic concentration of LPS (50 ng/mL). B cell proliferation was measured by [^3^H]-thymidine incorporation at 36 h by pulsing the cells overnight with 1 µCi/well. Uptake of [^3^H]-thymidine was quantified using a scintillation counter and expressed as mean cpm ± SD of triplicate wells. Results are representative of three independent experiments. (B) The percentage of CD86^+^ cells was measured 16 h after stimulation with 1 µg/ml HEL, gating on the B220^+^IgM^a+^ population.

Anergic B cells do not proliferate and demonstrate impaired induction of CD86 in response to antigenic stimulation [29], [30]. Therefore, sorted B cells were stimulated *in-vitro* with various concentrations of HEL together with a sub-mitogenic concentration of LPS. As shown in Figure 2A, B cells from both B6 and NZB IgTg mice showed a strong proliferative response to HEL in a concentration-dependent fashion. In contrast, neither B6 nor NZB dTg B cells proliferated in response to any of the concentrations of HEL tested, suggesting that NZB dTg B cells are equivalently anergic to their B6 counterparts. Consistent with this observation, induction of CD86 expression following overnight incubation with HEL was similarly reduced for B6 and NZB dTg B cells, as compared to corresponding IgTg controls (Figure 2B). Thus, B cells from NZB dTg mice are both phenotypically and functionally anergic.

### NZB dTg mice have an expansion of T2 cells

To investigate whether the breakdown of anergy in NZB dTg mice was accompanied by a failure to exclude anergic B cells from the marginal zone, we used anti-B220 and -CD21 in combination with anti-CD24 or -CD23 to define splenic B cell subsets. Although NZB IgTg mice have an increased proportion of marginal zone (CD21^hi^CD23^−^) B cells as compared to their B6 counterparts [23], the proportion of marginal zone and marginal zone precursor (CD21^hi^CD23^+^ or CD21^hi^CD24^hi^) cells were significantly reduced in NZB dTg mice comparably to B6 dTg mice (Table 1). These findings suggest that anergic B cells are appropriately excluded from the marginal zone in NZB mice. Consistent with this, immunofluorescence microscopy revealed no B220^+^HEL^+^ B cells within the marginal zone of NZB dTg mice (data not shown). Nevertheless, NZB dTg mice had an increased proportion of T2 (CD21^int^CD24^hi^) and follicular B cells (Fo, CD21^int^CD24^int^), which appeared to result from a shift towards a more mature phenotype within the transitional compartment (Table 1).

### NZB dTg B cells demonstrate enhanced survival following transfer into sHEL recipients

**Figure 3:**
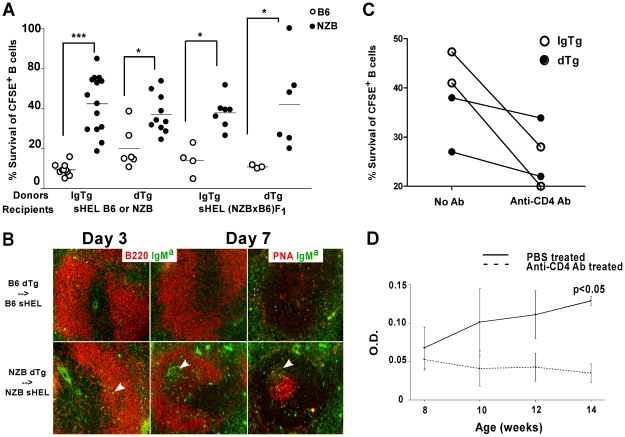
Increased survival of NZB dTg B cells following transfer into sHEL recipients. (A) Percent B220^+^ CFSE^+^ B cells surviving in sHEL recipient mice expressed as a percentage of survival in non-Tg mice, 3 d following transfer of T cell-depleted CFSE-labelled splenocytes from B6 or NZB, IgTg or dTg mice. Open circles indicate B6 mice and filled circles NZB mice, with horizontal lines representing the mean. Asterisks indicate the significance level as determined by the Mann-Whitney test: * p<0.05, ** p<0.005, *** p<0.0005. (B) Immunofluorescence microscopy of spleens from sHEL B6 or NZB mice, 3 or 7 d following transfer of T cell-depleted CFSE-labelled splenocytes from dTg mice. Sections were stained with anti-B220 or PNA, and anti-IgM^a^. Arrows indicate IgM^a+^ cells in the B cell follicle (d 3) or germinal center (d 7). (C) B cell survival following transfer into NZB sHEL recipient mice injected with PBS alone (No Ab) or anti-CD4 Ab. Open circles indicate NZB IgTg mice and filled circles NZB dTg mice. % differences in the survival of transferred cells due to CD4^+^ T cell depletion: NZB IgTg  = 20.17±1.18; NZB dTg  = 4.55±0.67. (D) Serum IgM^a^ anti-HEL Ab production following depletion of CD4^+^ T cells in NZB dTg mice. Mice were injected with anti-CD4 mAb or PBS beginning 8 wks of age and bled every 2 weeks until 14 wks of age. *P* values were calculated using a two way ANOVA followed by Bonferroni post-hoc analysis.

Although the expansion of T2 cells in NZB dTg mice occurred within a monoclonal repertoire, we questioned whether this might reflect a failure to exclude and/or delete anergic B cells. To address this possibility, we performed adoptive transfer experiments in which IgTg or dTg B cells were transferred into sHEL recipient mice. Freshly isolated T cell-depleted splenocytes were CFSE-labeled and the fate of the transferred cells determined by flow cytometry and immunofluorescence microscopy. Consistent with previous reports by ourselves and others, the majority (approximately 80–90%) of B6 IgTg or dTg B cells transferred into sHEL B6 were eliminated 3 days following transfer (Figure 3A) [14], [31]. In contrast, transferred NZB IgTg and dTg B cells demonstrated significantly enhanced survival, with 35–50% of the cells remaining on day 3. Immunofluorescence microscopy revealed that some of these surviving cells migrated into the B cell follicle (Figure 3B).

We have previously shown that NZB IgTg B cells not only survive but also proliferate and differentiate into anti-HEL Ab-producing cells within 3 days of being transferred into sHEL NZB recipient mice [31]. On day 7, Ab production was further augmented and accompanied by recruitment of IgTg B cells into germinal centers. This abnormal response resulted from an intrinsic defect in NZB B cells and was CD4^+^ T cell- and MHC-dependent. Unlike their IgTg counterparts, NZB dTg B cells failed to proliferate and did not differentiate into Ab-forming cells by day 3. In addition, their survival was less dependent on CD4^+^ T cells than their IgTg counterparts (Figure 3C). Nevertheless, by 7 days post transfer dTg B cells had been recruited into germinal centers, although at a considerably reduced extent than seen following transfer of IgTg B cells (Figure 3B). No surviving B6 dTg B cells could be detected at day 7. This suggests that immune mechanisms that lead to exclusion and elimination of anergic B cells from the peripheral repertoire are defective in NZB mice and that this permits anergic B cells to access T cell help, albeit inefficiently.

To further investigate the role of T cells in the breach of tolerance, young NZB dTg mice were given regular injections of purified anti-CD4 mAb to deplete CD4^+^ T cells, or PBS alone. Treatment with anti-CD4 mAb significantly reduced anti-HEL Ab production in NZB dTg mice (Figure 3D), suggesting that CD4^+^ T cell help contributes to the breach of tolerance in these mice. In support of this concept staining of spleen sections from NZB dTg mice revealed IgM^a+^HEL^+^-staining germinal centers (data not shown) which did not appear to result from activation of cells that co-expressed IgM^b^.

### Serum BAFF is significantly elevated in NZB mice and promotes NZB dTg B cell survival following adoptive transfer

**Figure 4:**
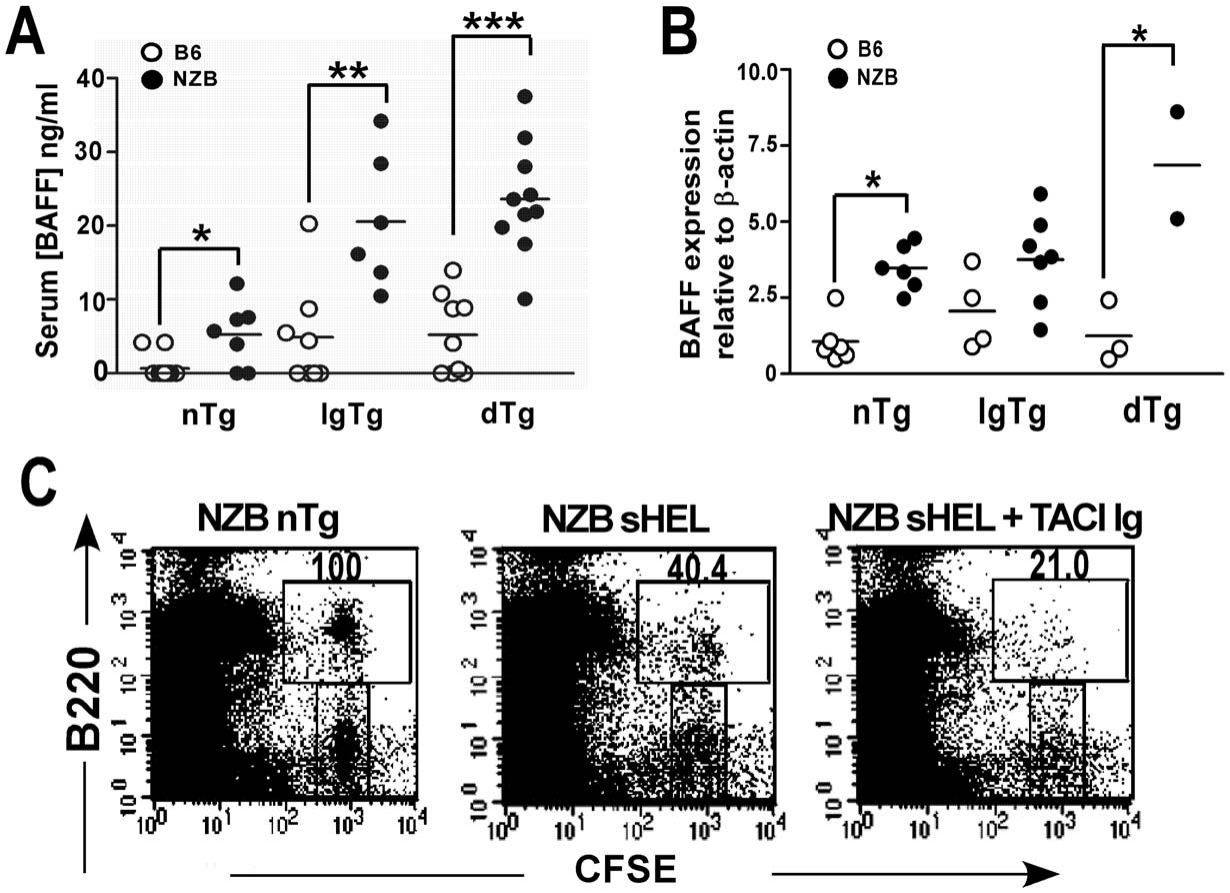
Elevated BAFF levels in NZB mice enhance survival of transferred NZB dTg B cells. (A) Serum BAFF levels in 6–12 wk old non-Tg (nTg), IgTg and dTg, B6 and NZB mice. (B) *Baff* mRNA expression in the splenocytes of 8–14 wk old non-Tg (nTg), IgTg and dTg, B6 and NZB mice. Horizontal lines represent the mean for each group examined. Significant p values for the difference between B6 and NZB mice are shown and were determined by one way ANOVA test (Kruskal-Wallis test) followed by Dunns' post test. (C) NZB sHEL recipient mice were injected with TACI-Ig or PBS alone, 1 d before transfer of CFSE-labelled NZB dTg B cells. Splenocytes were analyzed 3 d later by flow cytometry. Numbers indicate the percentage of surviving cells, determined by the ratio of CFSE^+^ B220^+^ cells to CFSE^+^B220^-^ cells as compared to a NZB non-Tg control. Asterisks indicate the significance level for comparison between B6 and NZB mice as determined by the Mann-Whitney test: * p<0.05, ** p<0.005, *** p<0.0005.

Since BAFF can rescue anergic self-reactive B cells from deletion by permitting their entry into the follicular and marginal zone B cell compartments [21], [32], we questioned whether NZB mice had elevated levels of BAFF. As shown in Figure 4A, 6–12 wk NZB non-Tg, IgTg, and dTg mice had elevated levels of BAFF as compared to their B6 counterparts. The increased levels of BAFF in NZB IgTg and dTg mice did not result from differences in the number of B cells, as splenic *baff* RNA expression was also significantly increased (Figure 4B).

To determine whether the increased survival of adoptively transferred NZB dTg B cells was BAFF-dependent, NZB sHEL recipient mice were injected with TACI-Ig, or PBS alone, 1 day before transfer of CFSE-labelled dTg B cells and were analyzed 3 days later. In 2 of 3 recipient mice, a single TACI-Ig injection resulted in significant depletion (>50%) of the marginal zone precursor and marginal zone B cell populations in recipient mice. In both of these mice, survival of transferred dTg B cells was reduced two-fold as compared to PBS-injected recipients (Figure 4C). Thus, the increased survival of NZB dTg B cells is BAFF-dependent.

### Heightened survival response of NZB B cells to BAFF

**Figure 5:**
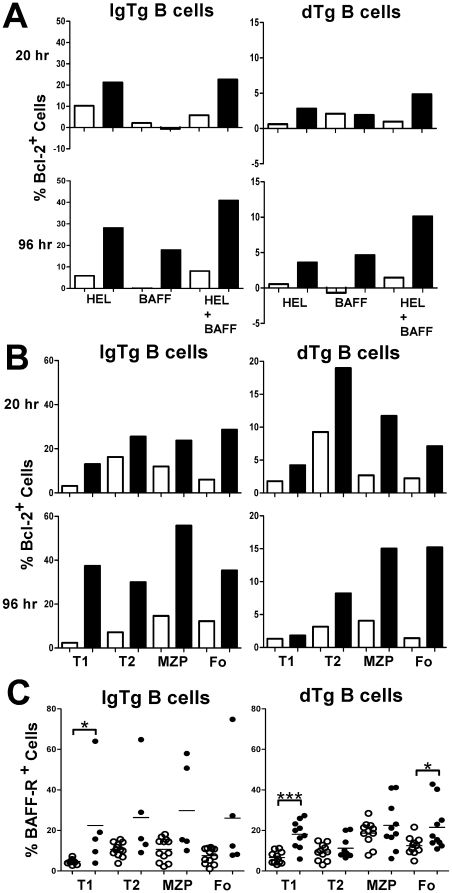
Heightened survival response of NZB dTg B cells to BAFF *in-vitro*. (A) Up-regulation of Bcl-2 in NZB IgTg and dTg B cells. B cells isolated from B6 (open bars) or NZB (filled bars) IgTg or dTg mice were incubated with media alone or media containing HEL (100 ng/ml), BAFF (40 ng/ml), or a combination of both, for 20 or 96 h at 37°C. The percent Bcl-2 positive cells was determined by flow cytometry gating on the B220^+^ population. Results shown are for a single mouse from each strain and are representative experiment of four independent experiments in which a total of 2 nTg, 5 IgTg, 7 dTg B6 mice, and 3 nTg, 6 IgTg, 4 dTg NZB mice were examined. (B) Enhanced expression of Bcl-2 in all B cell subsets of NZB IgTg or dTg B cells after incubation with HEL and BAFF. Cells were isolated and incubated for 96 h at 37°C, as outlined in (A). The percent Bcl-2 positive cells was determined by flow cytometry gating on the B220^+^ population and using CD21 and CD24 antibodies to subset B cells, as shown in Table 1. (C) Scatterplots showing the proportion of BAFF-R^+^ cells in various B cell subsets in B6 (open circles) and NZB (closed circles), IgTg and dTg mice, gated as in (B). Background staining with a relevant isotype control was extremely low, and similar in B6 and NZB mice. Asterisks indicate the significance level for comparison between B6 and NZB mice as determined by the Mann-Whitney test: * p<0.05, ** p<0.005, *** p<0.0005.

The increased survival of NZB dTg B cells following transfer into sHEL recipients was not solely due to increased levels of BAFF in the NZB environment, because NZB dTg B cells also demonstrated enhanced survival following transfer into sHEL (NZB x B6)F_1_ recipients (see Figure 3A). This finding raised the possibility that NZB dTg B cells have a heightened response to BAFF leading to their increased survival. Since BAFF has been shown to enhance B cell survival by at least two mechanisms: down-regulation of the pro-apoptotic molecule Bim [32], [33] and up-regulation of anti-apoptotic molecules such as Bcl-2 [15], [34], [35], we hypothesized that the increased survival of NZB dTg B cells results from altered expression of these molecules. To assess this possibility, B cells from B6 and NZB non-Tg, IgTg or dTg mice were stimulated with HEL in the presence or absence of BAFF for 20 hr and expression of Bim or Bcl-2 assessed using flow cytometry. Bim expression was unaffected by the presence or absence of BAFF or HEL for both B6 and NZB B cells at 20 hr (data not shown). Although incubation of NZB IgTg B cells with BAFF also did not result in significant changes in Bcl-2 expression at 20 hr, Bcl-2 expression was induced by incubation with HEL (Figure 5A). At 96 hr, Bcl-2 expression was significantly increased in IgTg B cells incubated with BAFF in the presence or absence of HEL (Figure 5A). Notably, NZB dTg B cells responded similarly to IgTg B cells with increased expression of Bcl-2 in response to HEL at 20 hr and increased expression of Bcl-2 in response to BAFF and HEL at 96 hr. Incubation of B6 dTg B cells with HEL and/or BAFF resulted in minimal changes in the expression of Bcl-2 at 20 or 96 hr. This was not due to the altered proportions of B cell subsets in NZB IgTg and dTg mice, because increased expression of Bcl-2 was seen in all peripheral B cell subsets (T1, T2, MZP and Fo) of these mice (Figure 5B). These findings suggest that the increased survival response of NZB dTg B cells results from altered expression of Bcl-2, but not Bim. Notably, there was a trend to increased expression of BAFF-R on all peripheral B cell populations in NZB IgTg and dTg mice, as compared to their B6 counterparts. This appeared to reflect an increased proportion of cells expressing BAFF-R rather than a shift in overall staining within these populations and did not arise from differences in BAFF binding between these mice, as staining with the anti-BAFF-R antibody is not affected by binding to BAFF. Thus, it is likely that the altered BAFF response in NZB dTg mice arises at least in part from increased BAFF-R expression.

## Discussion

In this study, we used a well-characterized transgenic model of B cell anergy to explore the mechanisms that lead to the breakdown of B cell tolerance in NZB mice. We show that although dTg B cells in NZB mice appear functionally and phenotypically anergic, they are recruited into germinal centers and/or differentiate into autoAb producing cells. Production of autoAbs in these mice was T cell-dependent. Similar to our previous findings for NZB IgTg B cells, NZB dTg B cells demonstrated increased survival following transfer into sHEL recipients. However, in contrast to NZB IgTg B cells, the increased survival of NZB dTg B cells was relatively T cell-independent and required BAFF. Although serum BAFF levels were elevated in NZB mice, the increased NZB dTg B cell survival appeared to arise from an altered response to BAFF resulting in increased expression of Bcl-2. Thus, BAFF and T cells cooperate to overcome tolerance in NZB dTg mice in the setting of abnormal B cell function.

While our findings suggest a role for BAFF in the breach of tolerance in NZB dTg mice they do not precisely recapitulate those in BAFF-Tg dTg mice. Whereas, dTg B cells from BAFF-Tg mice have an increased capacity to upregulate CD86 in response to HEL stimulation (21), NZB dTg B cells retain their anergic phenotype. This occurs despite evidence for increased maturation of B cells in NZB dTg mice, suggesting that entry into the mature B cell pool is insufficient to restore responsiveness to HEL in dTg B cells. In addition, production of autoAb in NZB dTg mice is T cell-dependent, while T cells are not required for the breach of tolerance in BAFF-Tg dTg mice. It is likely that these differences result from the considerably lower (5–10 fold) levels of BAFF in NZB as compared to BAFF-Tg mice (unpublished observations) together with the presence of additional signalling abnormalities in NZB dTg lymphocytes.

All of the B cell populations examined demonstrated increased expression of Bcl-2 following antigen-receptor engagement in the presence of BAFF *in-vitro*. We have previously shown that NZB mice have an increased proportion of T1 B cells that demonstrate features of Ag engagement and express high levels of Bcl-2. The findings reported herein suggest that altered BAFF signalling contributes to this phenotype and may act to enhance entry of self-reactive B cells into more mature B cell compartments. In support of this conclusion, the proportion of T2 and follicular B cells was increased in NZB dTg mice as compared to B6 controls and in our adoptive transfer experiments the majority of residual cells on day 3 following BAFF blockage were CD21^low^, indicating that BAFF is required to increase the survival of T2 and mature cells in these mice [36].

Studies suggest that over-expression of anti-apoptotic Bcl-2 family members, while enhancing cell survival, are not sufficient to overcome anergy [13], [37]. Therefore, it is probable that other immune abnormalities contribute to the breach of tolerance in NZB dTg mice. In NZB dTg mice, production of IgM anti-HEL Ab was accompanied by increased numbers of anti-HEL Ab-producing cells and expansion of late pre-plasma cells (CD138^high^) in the bone marrow. Since plasma cell differentiation of self-tolerant B cells is regulated at the early to late pre-plasma cell stage [24], our findings indicate that this tolerance checkpoint is defective in NZB mice. Given that anti-HEL Ab production is T cell-dependent in NZB dTg mice, it is likely that T cell signals play an important role in the generation of this phenotype. We have previously shown that NZB resting B cells are hyper-responsive to T cell signals, such as those delivered by CD40 and T cell cytokines [38]. Although anergic B cells have a block in BCR-mediated signal transduction, they retain the ability to respond to CD40L and T cell cytokines such as IL-4 [29]. Consequently, NZB B cell hyper-responsiveness to T cell-derived signals could allow the dTg B cells to respond to the limited T cell help available, resulting in their differentiation to the late pre-plasma stage, Ab-producing cells, and recruitment into germinal centers. It is likely that the increased proportion of T2 and follicular B cells in NZB dTg mice facilitates these interactions as studies show that T1 B cells do not respond efficiently to T cells [36]. Notably, chronic anti-CD4 treatment of NZB dTg mice resulted in a slight reduction in the proportion of CD21^int^ and enrichment in the proportion of CD21^low^ cells, suggesting that CD4^+^ T cells act to promote cell survival of the T2 and follicular B cell compartments similar to BAFF.

It is possible that the increased levels of BAFF and/or BAFF hyper-responsiveness, also contribute to the breach of B cell anergy in NZB dTg mice. BAFF has been shown to act as a survival factor for pre-plasma and plasma cells [39],[40], and thus could directly facilitate survival of late pre-plasma and Ab-producing cells in these mice. Additionally, as BAFF promotes survival of B cells during selection in germinal centers, the BAFF abnormalities in NZB dTg mice could prevent death of HEL-reactive B cells in the germinal centers [41], [42], augmenting T cell-dependent production of anti-HEL Ab in these mice [43], [44]. In support of the latter possibility, it is notable that several NZB dTg mice produced high levels of IgA anti-HEL Ab, which is augmented by BAFF, in the absence of significant amounts of IgG anti-HEL Ab, where BAFF plays a relatively minor role (unpublished observation) [45].

It has been suggested that a polymorphism in the promoter region of the *fcgr2b* gene that leads to reduced expression of FcγRIIb in germinal centre B cells, may contribute the breach of tolerance in NZB mice [46], [47]. Genetic manipulations that increase expression of FcγRIIb have been shown to reduce IgG autoantibody production in several other lupus-prone mouse models [48]. However, subsequent studies have indicated that this does not result from restoration of a generalized B cell tolerance defect, but instead from the effect of FcγRIIb on differentiation to and/or survival of IgG autoantibody producing plasmablasts [49], [50]. Our findings indicate that there is a more generalized breach of tolerance in NZB mice that impacts on survival of self-reactive B cells and their recruitment into the germinal centre and antibody forming compartments.

It is likely that our findings are relevant to human SLE. Although elevated levels of BAFF are seen some SLE patients [16]–[18], many patients have little or no elevation of serum BAFF. Our findings suggest that even in these patients, BAFF may play a role in breaching tolerance.

## Materials and Methods

### Ethics Statement

Mice were housed in a Canadian Council on Animal Care (CCAC) approved facility and all experiments were performed under the University Health Network Animal Care Committee approved protocol #123.

### Mice

NZB mice were purchased from Harlan-Sprague-Dawley (Blackthorne, England). C57BL/6 (B6) mice and B6 mice expressing transgenes encoding sHEL (ML5) or IgM/IgD heavy and light chains specific for HEL (MD4; IgTg) were purchased from The Jackson Laboratory (Bar Harbor, ME) [22]. Transgenes were backcrossed onto the NZB background using the speed congenic technique [51]. Double Tg (dTg) mice that expressed both Ig and sHEL transgenes were produced by intercrossing IgTg and sHEL mice. Mice were housed in specific pathogen free microisolators at the Toronto Western Hospital animal facility.

### ELISA and ELISpot Assays

Levels of anti-HEL IgM^a^ Ab and BAFF were measured by ELISA, using commercially available Ab (R&D Systems). Recombinant soluble mouse BAFF (Apotech, Switzerland) was used to generate a standard curve and sera from BAFF Tg mice (a generous gift from Dr. J. Gommerman) as a positive control. Ab-producing cells were detected by ELISpot, as previously described [31].

### Flow cytometry staining and analysis

Erythrocyte-depleted spleen or bone marrow cells were stained and analyzed as previously described [11]. The following directly conjugated mAbs were purchased from BD: biotin-conjugated anti-B220 (RA3-6B2), -IgM^a^ (DS-1), -CD24 (M1/69), PE-conjugated anti-IgM^a^ (DS-1), -CD23 (B3B4), -CD24 (M1/69), -CD138 (281-2), -B220, allophycocyanin-conjugated anti-CD19 (1D3), –CD21 (7G6), FITC-conjugated anti-CD21, and hamster IgG controls. Biotinylated polyclonal rabbit anti-HEL Ab was purchased from Rockland (Gilbertsville, PA), FITC-anti-BAFF-R from R&D, and all isotype controls from Caltag. Allophycocyanin- or PerCP-conjugated streptavidin (BD) was used to reveal biotinylated Ab staining.

### Immunofluorescent staining of tissue sections

Cryostat spleen sections (5 µm) were fixed and stained as previously described [31]. Tissue fluorescence was visualized using a Zeiss Axioplan 2 imaging microscope (Oberkochen, Germany).

### *In-vitro* assays of B cell proliferation and CD86 upregulation

B220^+^ splenic cells were sorted using a MoFlow instrument (Cytomation Inc., CO). For proliferation assays, 5×10^4^ B cells were cultured in triplicate in media alone or a submitogenic concentration of LPS (50 ng/ml) with various concentrations of HEL (0–1000 ng/ml). B cell proliferation was measured by [^3^H]-thymidine incorporation. For induction of CD86 expression, 5×10^5^ sorted B cells or T cell-depleted splenocytes from IgTg or dTg mice were incubated in culture media alone or containing HEL (1 µg/ml) at 37°C overnight. Cultured cells were stained with anti-B220, -IgM^a^, and -CD86 mAb.

### Measurement of intracellular Bim and Bcl-2 expression

B cells were purified from the spleens of non-Tg, IgTg or dTg mice by negative selection using a Dynal Mouse B cell Negative Isolation Kit (Invitrogen), and incubated at 37°C for 20 or 96 hr in media alone or containing HEL (100 ng/ml), BAFF (40 ng/ml) or a combination of HEL and BAFF. Cells were stained with anti-B220 or -IgM^a^, -CD21, -CD24, and PE-conjugated anti-BCL-2 (3F11;BD) or hamster control (A19-3;BD) Ab to assess Bcl-2 expression, or anti-CD19 and rat anti-mouse Bim (eBioscience), followed by allophycocyanin-conjugated anti-rat IgG (BD) FITC to assess Bim, The cells were fixed and permeabilized prior to intracellular staining using Cytofix/Cytoperm (BD).

### Adoptive transfers

Splenocytes were depleted of T cells and labelled with CFSE (Molecular Probes) prior to transfer, as previously described [31]. To block BAFF, recipient mice were given as single intra-peritoneal injection with 160 µg TACI-Ig (R&D) or PBS one day before adoptive transfer. To deplete CD4^+^ T cells, recipient mice were given two intra-peritoneal injections with 0.5 mg anti-CD4 Ab or PBS 5 days and one day before donor cell transfer.

### CD4^+^ cell depletion

Anti-CD4 mAb was purified as previously described [31]. Starting at 5–6 weeks of age NZB dTg mice were given two intra-peritoneal injections with 0.5 mg anti-CD4 Ab or PBS spaced 3 days apart and this treatment was repeated every 3 weeks. This was sufficient to deplete >95% of CD4^+^ T cells.

### BAFF mRNA expression

RNA was purified from 10–14 wk old mice using an RNeasy Mini Kit (Qiagen, Switzerland), treated with DNAseI (Invitrogen), and reverse transcribed into cDNA (Applied Biosystems, CA). Quantitative real-time PCR was performed with SYBR Green Master Mix on an ABI/PRISM 7900 HT sequence detector system (Applied Biosystems). Primer sequences were designed to span exon-to-exon for beta actin (β-actin, TTGCTGACAGGATGCAGAAG and GTACTTGCGCTCAGGAGGAG) and BAFF (TTCCATGGCTTCTCAGCTTT and CGTCCCCAAAGACGTGTACT). Gene expression was analyzed using the relative standard curve method where BAFF expression was normalized to β-actin.

### Statistics

Comparisons of differences between groups of mice were performed using the Mann-Whitney non-parametric test, or a two way ANOVA followed with Bonferroni post-hoc analysis as indicated in figure legends.

## References

[1] Theofilopoulos AN, Dixon FJ (1985). Murine models of systemic lupus erythematosus.. Adv Immunol.

[2] Izui S, McConahey PJ, Dixon FJ (1978). Increased spontaneous polyclonal activation of B lymphocytes in mice with spontaneous autoimmune disease.. J Immunol.

[3] Wither JE, Roy V, Brennan LA (2000). Activated B cells express increased levels of costimulatory molecules in young autoimmune NZB and (NZB x NZW)F(1) mice.. Clin Immunol.

[4] Manny N, Datta SK, Schwartz RS (1979). Synthesis of IgM by cells of NZB and SWR mice and their crosses.. J Immunol.

[5] Moutsopoulos HM, Boehm-Truitt M, Kassan SS, Chused TM (1977). Demonstration of activation of B lymphocytes in New Zealand black mice at birth by an immunoradiometric assay for murine IgM.. J Immunol.

[6] Raveche ES, Steinberg AD, DeFranco AL, Tjio JH (1982). Cell cycle analysis of lymphocyte activation in normal and autoimmune strains of mice.. J Immunol.

[7] Wangel AG, Milton A, Egan JB (1982). Spontaneous plaque forming cells in the peripheral blood of patients with systemic lupus erythematosus.. Clin Exp Immunol.

[8] Goodnow CC, Cyster JG, Hartley SB, Bell SE, Cooke MP (1995). Self-tolerance checkpoints in B lymphocyte development.. Adv Immunol.

[9] Halverson R, Torres RM, Pelanda R (2004). Receptor editing is the main mechanism of B cell tolerance toward membrane antigens.. Nat Immunol.

[10] Hippen KL, Schram BR, Tze LE, Pape KA, Jenkins MK (2005). In vivo assessment of the relative contributions of deletion, anergy, and editing to B cell self-tolerance.. J Immunol.

[11] Roy V, Chang NH, Cai Y, Bonventi G, Wither J (2005). Aberrant IgM signaling promotes survival of transitional T1 B cells and prevents tolerance induction in lupus-prone New Zealand black mice.. J Immunol.

[12] Hartley SB, Cooke MP, Fulcher DA, Harris AW, Cory S (1993). Elimination of self-reactive B lymphocytes proceeds in two stages: arrested development and cell death.. Cell.

[13] Fang W, Weintraub BC, Dunlap B, Garside P, Pape KA (1998). Self-reactive B lymphocytes overexpressing Bcl-xL escape negative selection and are tolerized by clonal anergy and receptor editing.. Immunity.

[14] Cyster JG, Hartley SB, Goodnow CC (1994). Competition for follicular niches excludes self-reactive cells from the recirculating B-cell repertoire.. Nature.

[15] Batten M, Groom J, Cachero TG, Qian F, Schneider P (2000). BAFF mediates survival of peripheral immature B lymphocytes.. J Exp Med.

[16] Cheema GS, Roschke V, Hilbert DM, Stohl W (2001). Elevated serum B lymphocyte stimulator levels in patients with systemic immune-based rheumatic diseases.. Arthritis Rheum.

[17] Stohl W, Metyas S, Tan SM, Cheema GS, Oamar B (2003). B lymphocyte stimulator overexpression in patients with systemic lupus erythematosus: longitudinal observations.. Arthritis Rheum.

[18] Zhang J, Roschke V, Baker KP, Wang Z, Alarcon GS (2001). Cutting edge: a role for B lymphocyte stimulator in systemic lupus erythematosus.. J Immunol.

[19] Khare SD, Sarosi I, Xia XZ, McCabe S, Miner K (2000). Severe B cell hyperplasia and autoimmune disease in TALL-1 transgenic mice.. Proc Natl Acad Sci U S A.

[20] Mackay F, Woodcock SA, Lawton P, Ambrose C, Baetscher M (1999). Mice transgenic for BAFF develop lymphocytic disorders along with autoimmune manifestations.. J Exp Med.

[21] Thien M, Phan TG, Gardam S, Amesbury M, Basten A (2004). Excess BAFF rescues self-reactive B cells from peripheral deletion and allows them to enter forbidden follicular and marginal zone niches.. Immunity.

[22] Goodnow CC, Crosbie J, Adelstein S, Lavoie TB, Smith-Gill SJ (1988). Altered immunoglobulin expression and functional silencing of self-reactive B lymphocytes in transgenic mice.. Nature.

[23] Roy V, Bonventi G, Cai Y, MacLeod R, Wither JE (2007). Immune mechanisms leading to abnormal B cell selection and activation in New Zealand Black mice.. Eur J Immunol.

[24] Culton DA, O'Conner BP, Conway KL, Diz R, Rutan J (2006). Early preplasma cells define a tolerance checkpoint for autoreactive B cells.. J Immunol.

[25] Fulcher DA, Basten A (1994). Reduced life span of anergic self-reactive B cells in a double-transgenic model.. J Exp Med.

[26] Fulcher DA, Lyons AB, Korn SL, Cook MC, Koleda C (1996). The fate of self-reactive B cells depends primarily on the degree of antigen receptor engagement and availability of T cell help.. J Exp Med.

[27] Cornall RJ, Cyster JG, Hibbs ML, Dunn AR, Otipoby KL (1998). Polygenic autoimmune traits: Lyn, CD22, and SHP-1 are limiting elements of a biochemical pathway regulating BCR signaling and selection.. Immunity.

[28] Inaoki M, Sato S, Weintraub BC, Goodnow CC, Tedder TF (1997). CD19-regulated signaling thresholds control peripheral tolerance and autoantibody production in B lymphocytes.. J Exp Med.

[29] Cooke MP, Heath AW, Shokat KM, Zeng Y, Finkelman FD (1994). Immunoglobulin signal transduction guides the specificity of B cell-T cell interactions and is blocked in tolerant self-reactive B cells.. J Exp Med.

[30] Rathmell JC, Fournier S, Weintraub BC, Allison JP, Goodnow CC (1998). Repression of B7.2 on self-reactive B cells is essential to prevent proliferation and allow Fas-mediated deletion by CD4(+) T cells.. J Exp Med.

[31] Chang NH, MacLeod R, Wither JE (2004). Autoreactive B cells in lupus-prone New Zealand black mice exhibit aberrant survival and proliferation in the presence of self-antigen in vivo.. J Immunol.

[32] Lesley R, Xu Y, Kalled SL, Hess DM, Schwab SR (2004). Reduced competitiveness of autoantigen-engaged B cells due to increased dependence on BAFF.. Immunity.

[33] Craxton A, Draves KE, Gruppi A, Clark EA (2005). BAFF regulates B cell survival by downregulating the BH3-only family member Bim via the ERK pathway.. J Exp Med.

[34] Do RK, Hatada E, Lee H, Tourigny MR, Hilbert D (2000). Attenuation of apoptosis underlies B lymphocyte stimulator enhancement of humoral immune response.. J Exp Med.

[35] Hsu BL, Harless SM, Lindsley RC, Hilbert DM, Cancro MP (2002). Cutting edge: BLyS enables survival of transitional and mature B cells through distinct mediators.. J Immunol.

[36] Meyer-Bahlburg A, Andrews SF, Yu KO, Porcelli SA, Rawlings DJ (2008). Characterization of a late transitional B cell population highly sensitive to BAFF-mediated homeostatic proliferation.. J Exp Med.

[37] Mandik-Nayak L, Nayak S, Sokol C, Eaton-Bassiri A, Madaio MP (2000). The origin of anti-nuclear antibodies in bcl-2 transgenic mice.. Int Immunol.

[38] Jongstra-Bilen J, Vukusic B, Boras K, Wither JE (1997). Resting B cells from autoimmune lupus-prone New Zealand Black and (New Zealand Black x New Zealand White)F1 mice are hyper-responsive to T cell-derived stimuli.. J Immunol.

[39] Avery DT, Kalled SL, Ellyard JI, Ambrose C, Bixler SA (2003). BAFF selectively enhances the survival of plasmablasts generated from human memory B cells.. J Clin Invest.

[40] O'Connor BP, Raman VS, Erickson LD, Cook WJ, Weaver LK (2004). BCMA is essential for the survival of long-lived bone marrow plasma cells.. J Exp Med.

[41] Hase H, Kanno Y, Kojima M, Hasegawa K, Sakurai D (2004). BAFF/BLyS can potentiate B-cell selection with the B-cell coreceptor complex.. Blood.

[42] Rahman ZS, Manser T (2005). Failed up-regulation of the inhibitory IgG Fc receptor Fc gamma RIIB on germinal center B cells in autoimmune-prone mice is not associated with deletion polymorphisms in the promoter region of the Fc gamma RIIB gene.. J Immunol.

[43] Sasaki Y, Casola S, Kutok JL, Rajewsky K, Schmidt-Supprian M (2004). TNF family member B cell-activating factor (BAFF) receptor-dependent and -independent roles for BAFF in B cell physiology.. J Immunol.

[44] Shulga-Morskaya S, Dobles M, Walsh ME, Ng LG, Mackay F (2004). B cell-activating factor belonging to the TNF family acts through separate receptors to support B cell survival and T cell-independent antibody formation.. J Immunol.

[45] Groom JR, Fletcher CA, Walters SN, Grey ST, Watt SV (2007). BAFF and MyD88 signals promote a lupuslike disease independent of T cells.. J Exp Med.

[46] Jiang Y, Hirose S, Sanokawa-Akakura R, Abe M, Mi X (1999). Genetically determined aberrant down-regulation of FcgammaRIIB1 in germinal center B cells associated with hyper-IgG and IgG autoantibodies in murine systemic lupus erythematosus.. Int Immunol.

[47] Xiu Y, Nakamura K, Abe M, Li N, Wen XS (2002). Transcriptional regulation of Fcgr2b gene by polymorphic promoter region and its contribution to humoral immune responses.. J Immunol.

[48] McGaha TL, Sorrentino B, Ravetch JV (2005). Restoration of tolerance in lupus by targeted inhibitory receptor expression.. Science.

[49] Brownlie RJ, Lawlor KE, Niederer HA, Cutler AJ, Xiang Z (2008). Distinct cell-specific control of autoimmunity and infection by FcgammaRIIb.. J Exp Med.

[50] Xiang Z, Cutler AJ, Brownlie RJ, Fairfax K, Lawlor KE (2007). FcgammaRIIb controls bone marrow plasma cell persistence and apoptosis.. Nat Immunol.

[51] Wakeland E, Morel L, Achey K, Yui M, Longmate J (1997). Speed congenics: a classic technique in the fast lane (relatively speaking).. Immunol Today.

